# A high*-*quality genome assembly and annotation of *Quercus acutissima* Carruth

**DOI:** 10.3389/fpls.2022.1068802

**Published:** 2022-11-24

**Authors:** Dan Liu, Xiaoman Xie, Boqiang Tong, Chengcheng Zhou, Kai Qu, Haili Guo, Zhiheng Zhao, Yousry A. El-Kassaby, Wei Li, Wenqing Li

**Affiliations:** ^1^ National Engineering Research Center of Tree Breeding and Ecological Restoration, State Key Laboratory of Tree Genetics and Breeding, College of Biological Sciences and Technology, Beijing Forestry University, Beijing, China; ^2^ Shandong Provincial Center of Forest and Grass Germplasm Resources, Jinan, China; ^3^ Department of Forest and Conservation Sciences, The University of British Columbia, Vancouver, BC, Canada

**Keywords:** *Quercus acutissima*, genome assembly, gene annotation, phylogenetic analysis, gene families

## Abstract

**Introduction:**

*Quercus acutissima* is an economic and ecological tree species often used for afforestation of arid and semi-arid lands and is considered as an excellent tree for soil and water conservation.

**Methods:**

Here, we combined PacBio long reads, Hi-C, and Illumina short reads to assemble *Q. acutissima* genome.

**Results:**

We generated a 957.1 Mb genome with a contig N50 of 1.2 Mb and scaffold N50 of 77.0 Mb. The repetitive sequences constituted 55.63% of the genome, among which long terminal repeats were the majority and accounted for 23.07% of the genome. *Ab initio*, homology-based and RNA sequence-based gene prediction identified 29,889 protein-coding genes, of which 82.6% could be functionally annotated. Phylogenetic analysis showed that *Q. acutissima* and *Q. variabilis* were differentiated around 3.6 million years ago, and showed no evidence of species-specific whole genome duplication.

**Conclusion:**

The assembled and annotated high-quality *Q. acutissima* genome not only promises to accelerate the species molecular biology studies and breeding, but also promotes genome level evolutionary studies.

## Introduction

As one of the largest genera in Fagaceae, *Quercus* (oak) contains more than 400 widely distributed species in Asia, Europe, Africa, and North America (Simeone et al., 2016). Oaks have various utilities, including timber, bioenergy, and dyes production (Sasaki et al., 2014; Wu et al., 2014; Li et al., 2018). According to molecular classification, the genera *Quercus* has been divided into two subgenera, *Quercus* and *Cerris* (Denk and Grimm, 2010; Denk et al., 2017; Deng et al., 2018; Hipp et al., 2018). The subgenera *Quercus* includes five groups (sections): *Ponticae*, *Virentes*, *Protobalanus* (intermediate Oak), *Quercus* (white oak), and *Lobatae* (red oak), while *Cerris* includes three groups (sections): *Ilex*, *Cerris* and *Cyclobalanopsis* (Denk et al., 2017). Within the *Quercus* genera, the evolutionary profiles of plastid genomes have been elucidated in *Q. acutissima*, *Q. aliena*, *Q. aquifolioides*, *Q. baronii*, *Q. dolicholepis*, *Q. edithiae*, *Q. fabri*, *Q. glauca*, and 10 other *Quercus* plastomes (Li et al., 2021). However, only four species with whole genome sequences have been published, including *Q. lobata* (Sork et al., 2016), *Q. suber* (Ramos et al., 2018), *Q. robur* (Plomion et al., 2016), and *Q. acutissima* (Fu et al., 2022). Although the genome data of *Q. acutissima* have been published, the continuity of the assembly still needs improvement (Fu et al., 2022).

As an important ecological and economic tree species, *Q. acutissima* Carruth is widely distributed in East Asia, especially in southeast China (18° - 41° N, 91° - 123°E) (Li et al., 2018; Yang et al., 2019). The silvics of *Q. acutissima* is usually mixed or secondary monocultures, which are also distributed in a scattered manner in harsh environments (Aldrich et al., 2003; Zhang et al., 2013). *Q. acutissima* timber provides excellent building material and charcoal production in many Asian countries, including China, Japan, and Korea (Zhang et al., 2013). At present, research on *Q. acutissima* is mainly focused on propagation, eco-physiology, selection, and genetic diversity (Dong, 2008; Wang et al., 2009; Liao, 2012; Zhang et al., 2013). In northern China, *Q. acutissima* forest ecosystems have been degraded due to human disturbance, threatening the species genetic resources (Aldrich et al., 2003; Zhang et al., 2013). Thus, planning breeding and conservation programs for *Q. acutissima* native populations is crucial, and the understanding of the species genome-wide evolution, gene function, and molecular breeding are important elements to supporting these goal (Greene and Morris, 2001).

Here, the *Q. acutissima* genome was sequenced and *de novo* assembled using PacBio long reads, Hi-C reads, and Illumina short reads. We performed structural gene annotation, repetitive sequences identification, and executed comparative genomics with other plant genomes. Our results are expected to improve our understanding of the evolution and diversification of genes in *Q. acutissima*, laying the foundation for novel genes discovery and ultimately contributing to the development of novel properties for the species breeding programs.

## Materials and methods

### Plant materials, DNA extraction and genome sequencing

Fresh *Q. acutissima* leaves were collected from a tree growing in the Shandong Provincial Center of Forest and Grass Germplasm Resources (36.62°N, 117.16°E), immediately frozen in liquid nitrogen, and stored at -80°C until further use. Plant specimens (barcode number SDF1001228) and total genomic DNA (code ld001qa001) were stored in Shandong Provincial Center of Forest and Grass Germplasm Resources. Total genomic DNA was extracted from leaf tissue using the DNeasy Plant Mini Kit (Qiagen, Hilden, Germany) following the manufacturer’s instructions. After obtaining high-quality purified genomic DNA samples, PCR free SMRT bell library was constructed and sequenced by PacBio sequencing platform, and we obtained 154.41 Gb of subreads with 160× coverage. We also constructed a Hi-C library and a paired-end library with an insert size of 350 bp and sequenced using the Illumina HiSeq X Ten platform.

### Genome assembly, quality evaluation, and construction of pseudomolecule chromosomes

Before *Q. acutissima* genome *de novo* assembly, we used high-quality Illumina paired-end reads to estimate the genome size and heterozygosity with genomescope software (Vurture et al., 2017). Four software, including Canu (v2.1.1, default parameters) (Koren et al., 2017), FALCON (Chin et al., 2016), SmartDenovo (Istace et al., 2017), and WTDBG (Ruan and Li, 2019) were used to perform preliminary assembly of the genome. After the assembly of the third generation subreads, due to the presence of sequencing errors, a certain amount of error information existed such as short insertion-deletion mutations (Indel) and single-nucleotide polymorphism (SNP). Thus, we used the Illumina sort reads to polish this genome with BWA (v0.7.9a, parameter, -k 30) (Li and Durbin, 2009), and Pilon software (v1.22, default parameters) (Walker et al., 2014). Additionally, based on the OrthoDB (Kriventseva et al., 2019) database, we performed a BUSCO (version 3.0.1, default parameters) (Simão et al., 2015) assessment using single-copy orthologous genes to confirm the genome assembly quality. Quality control of the alignment reads was performed using the Phase Genomics Hi-C alignment quality control tool and scaffolding was carried out with Phase Genomics Proximo Hi-C genome scaffolding platform to obtain chromosome-level assembly.

### Genome annotation

We used a combination of *de novo* prediction and homology-based searches to annotate the genome tandem and interspersed repeats. First, RepeatModeler software (Flynn et al., 2020) was used to build the *de novo* repeat sequence library, and then we used RepeatMasker (Tarailo-Graovac and Chen, 2009), and Tandem Repeat Finder (Gary, 1999) software for repeat sequences prediction. Second, based on Repbase (Jurka et al., 2005), we used RepeatMasker to search homologous repeat sequences.

After repetitive sequence masking, we used three methods to predict gene structure. First, homology prediction was conducted by comparing homologous proteins from plant genomes, including *Q. lobata* (Sork et al., 2016), *Q. suber* (Ramos et al., 2018), *Q. robur* (Plomion et al., 2016), *Fagus sylvatica* (Mishra et al., 2018), and *Casuarina equisetifolia* (Ye et al., 2018) using Blast v2.2.28 and the GeneWise web resource v2.2.0 (Birney et al., 2004). Second, we used Augustus (Stanke et al., 2004), SNAP (https://github.com/KorfLab/SNAP), and GeneMark (Ter-Hovhannisyan et al., 2008) to *ab initio* gene prediction. Third, the PASA software (Roberts et al., 2011) was used to predict gene structure by aligning EST/cDNA sequences with the genome. Combining the above results, using the evincemodeler (EVM) (Haas et al., 2008) to integrate the gene set predicted by the three strategies into a nonredundant and more complete gene set.

We used the NCBI protein database, GO (Mi et al., 2019), KEGG (release 84.0) (Kanehisa et al., 2016), NR (ftp://ftp.ncbi.nlm.nih.gov/blast/db/FASTA/nr.gz), PFAM (Finn et al., 2014), and eggNOG-mapper (Cantalapiedra et al., 2021) to annotate gene function. The *E*-value cutoff was set to 1e-5 for BLAST searches.

### Gene families and phylogenetic analysis

We downloaded (https://www.ncbi.nlm.nih.gov/) and performed a comparative genomic investigation of *Q. acutissima* with *Q. robur*, *Q. mongolica*, *Q. lobata*, *Q. variabilis*, *Q. suber*, *Castanea mollissima*, *Castanea crenata*, *Castanopsis tibetana*, *Fagus sylvatica*, *Juglans regia*, *Cyclocarya paliurus*, *Carya illinoinensis*, *Morella rubra*, *Corylus mandshurica*, *Carpinus viminea*, *Betula pendula*, and *Vitis vinifera*. The software OrthoFinder2 v2.3.1 (Emms and Kelly, 2019) was used to identify homoeologous gene clusters. IQ-TREE v1.6.7 (Nguyen et al., 2015) was used to construct a phylogenetic tree based on single copy homoeologous genes. The MAFFT v7.4.07 (Katoh and Standley, 2013) was used to align homoeologs before transforming aligned protein sequences into codon alignment. The concatenated amino acid sequences were trimmed using trimAL v1.4 (Capella-Gutiérrez et al., 2009) with -gt 0.8 -st 0.001 -cons 60. Divergence times were estimated using the MCMCTree software (Yang, 2007) in the PAML v4.9h (Guindon et al., 2010) package with the BRMC method (Sanderson, 2003; Blanc and Wolfe, 2004), and the correction times were taken from the TimeTree (Kumar et al., 2017): 109.0-123.5 MYA split time between *V. vinifera* and *B. pendula*, 56.8-95.0 MYA split time between *Q. suber* and *B. pendula*, and 35.7-83.5 MYA split time between *J. regia* and *B. pendula.* Based on the clustering analysis of gene families and dating, gene family expansion and contraction analyses were performed using CAFÉ (De Bie et al., 2006).

### Synteny and WGD analysis

Syntenic blocks containing at least five genes were identified using the python version of MCScan (Huang et al., 2009; Schmutz et al., 2010) between *Q. mongolica*, *Q. variabilis*, *Q. acutissima*, *C. mollissima*, and *C. tibetana*. Genome circular plot was produced using Circos (Krzywinski et al., 2009). KaKs_Calculator 2.0 (Wang et al., 2010) was used to calculate *Ka, Ks*, and the *Ka/Ks* ratio by implementing the YN model.

### GO enrichment analysis

GO enrichment analysis was performed using the R package clusterProfiler (Yu et al., 2012). The *p* values were adjusted for multiple comparisons using the method of Benjamini and Hochberg (*p* < 0.05 was considered significant).

## Results

### Genome sequencing and assembly

**Figure 1:**
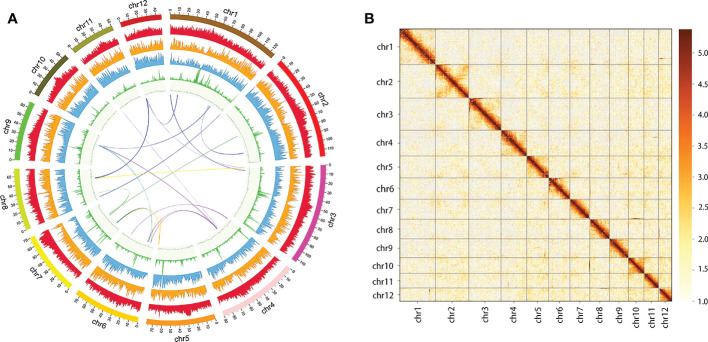
*Q. acutissima* genome features. **(A)** The genome circle plot (from the outer circle to the inner one, Class I transposable element (TE) density, Class II TE density, coding gene density, tandem repeat percentage, guanine-cytosine (GC) content, and co-linear block, respectively). **(B)** Twelve pseudo-molecules scaffolding with Hi-C data.

We sequenced Q. acutissima genome and generated a total of 154.41 Gb PacBio long reads with N50 of 24,256 bp (**Table S1**). The genome size and heterozygosity were estimated to be 750 Mb and 2.77% using K-mer analysis, respectively (**Figure S1**). To accurately assemble the Q. acutissima genome, we compared multiple assembly strategies in the primary step, and based on contiguity metrics including the total number of assembled contigs, N50, contigs’ maximum length, and the best assembly from Canu was selected for further polishing and scaffolding with Hi-C data. The assembled genome size was 957.09 Mb, including 1,507 contigs with an N50 length of 1.20 Mb and 15 scaffolds with N50 length 77.04 Mb (**Table 1**). The longest 12 scaffolds correspond to 12 pseudo-chromosomes (**Figure 1**).

**Table 1:** *Quercus acutissima* genome assembly statistics.

Assembly features		
Number of contigs	1,507
Contig N50 (Mb)	1.20
Number of scaffolds	15
Scaffold N50 (Mb)	77.04
Number of genes	29,889
Average gene length (bp)	4,476.10
Average exons per gene	4.92
Average exon length (bp)	253.60
Average intron length (bp)	824.50
Average Coding sequences length(bp)	1,247.79
Total size of repeat sequences (Mb)	532.33

### Assessment of genomic integrity

The completeness and accuracy of the genome assembly were evaluated using BUSCO. The high BUSCO complete ratio (98.00%) corroborated the genome assembly excellent quality (**Table S2**). The guanine*-*cytosine (GC) depth analysis showed that there was no obvious left-right chunking in the GC-depth plot (**Figure S2**) and the average GC content was 35.18% (**Table S3**). Approximately 99.84% of the Illumina short reads could be successfully mapped to the genome assembly (**Figure S3**, **Table S4**). These results suggest that the assembly of the *Q. acutissima* genome is highly accurate and continuous.

### Genome annotation

Through an integrative approach, we identified 546.67 Mb repetitive sequences, accounting for 57.13% of genome (**Table 1**, **Table S5**). The Long terminal repeat retrotransposons (LTR-RTs) from the largest proportion (23.07%) of the repeat (**Table S6**).

A total of 29,889 protein-coding genes were identified, their average lengths and coding sequences were 4,476.10 and 1,247.79 bp, respectively (**Table 1**). Based on the comparison between predicted gene sets with the annotation databases, a total of 24,689 (82.6%) genes were functionally annotated (**Table S7**).

### Gene family and phylogenetic relationships

**Figure 2:**
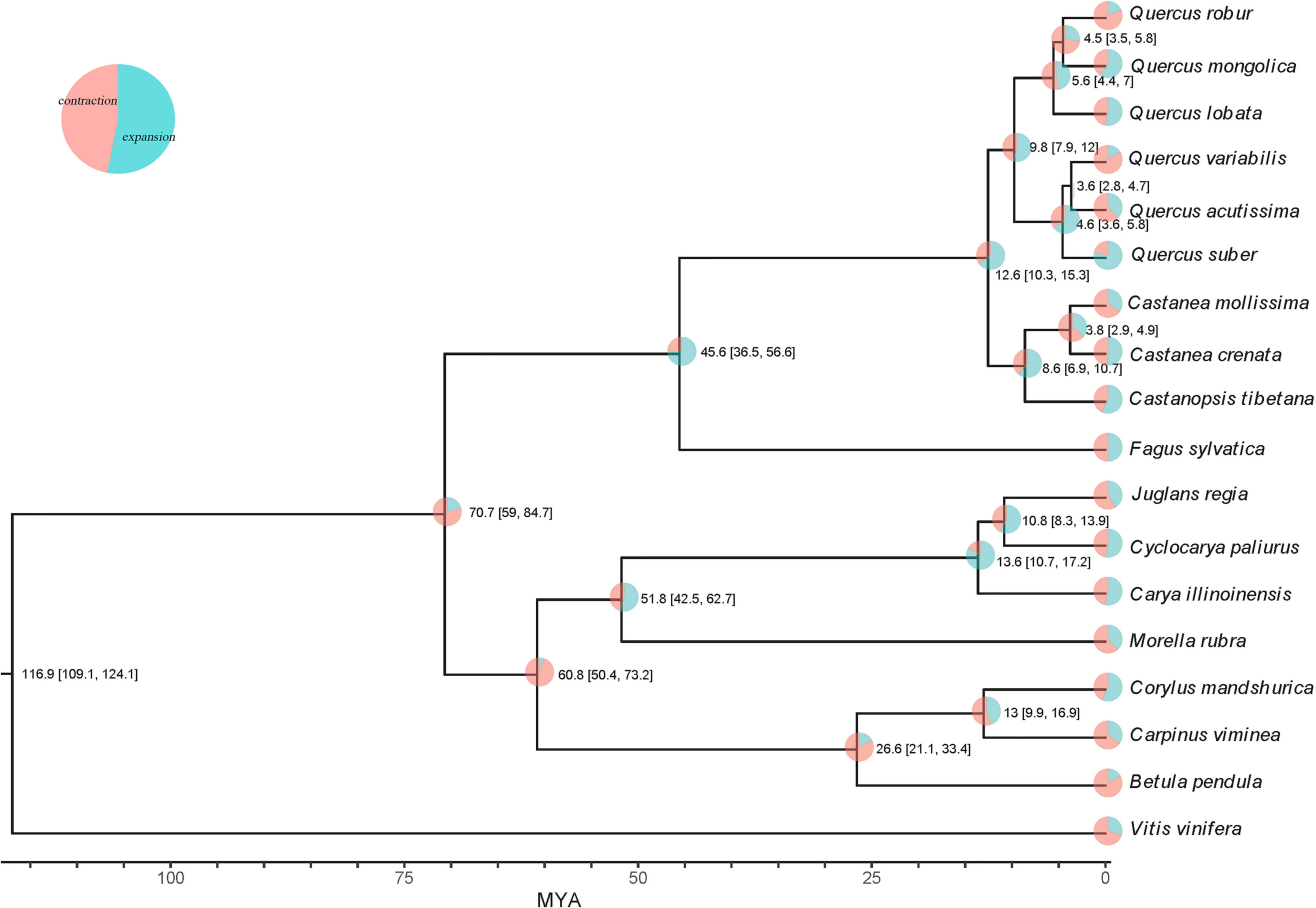
Maximum likelihood phylogenetic tree and expanded and contracted gene families in *Q. acutissima*. The numbers at the branch node in the tree indicate the divergence time and 95% confidence interval.

To assess the palaeohistory of *Q. acutissima*, we performed comparative genomic analyses incorporating *Q. acutissima* along with 16 other genomes and one outgroup (*V. vinifera*) (**Figure 2**). Out of the 28,312 gene families, only 10 were found to be unique to the *Q. acutissima* genome, and fewer than 60 gene families were unique to other *Quercus* (**Table S8**). Construction of the phylogenetic tree confirmed the evolutionary relationship within *Quercus*, and the divergence between *Q. variabilis* and *Q. acutissima* was estimated at 3.6 MYA (**Figure 2**). Expanded gene families provide the raw material for adaptation and trait evolution. We then examined the rates and direction of change in gene family size among taxa using CAFE (Han et al., 2013). The results showed that *Q. acutissima* exhibited larger numbers of contracted gene families (2,390) than expanded (3,897) (**Table S9**, **Figure 2**). These expanded families are mainly related to ion transport, such as ion transport, ion transmembrane transport, inorganic ion transmembrane transport (**Table S10**), while the contracted gene families were mainly enriched to glycosinolate biosynthetic process, sesquiterpene metabolic and biosynthetic process, monoterpenoid metabolic and biosynthetic process (**Table S11**).

### Whole-genome duplication and synteny analysis

**Figure 3:**
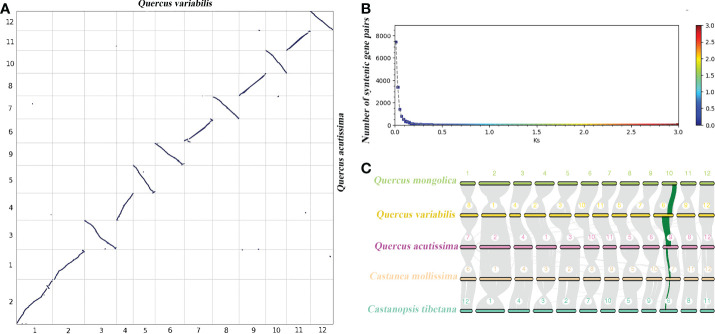
Syntenic dot plot and synteny analysis between *Q. acutissima* and other evaluated species. **(A)** Syntenic dot plot between the *Q. acutissima* and *Q. variabilis* genome. **(B)** Ks distribution between the *Q. acutissima* and *Q. variabilis* genome. **(C)** Synteny analyses among the genomes of *Q. acutissima, Q. mongolica, Q. variabilis, C. mollissima* and *C. tibetana.* Synteny blocks between paired chromosomes are connected by gray lines; one representative orthologous block (green lines) is noted.

Whole genome duplication (WGD) events are widespread and play a vital role in plant genome adaptation and evolution (Xue et al., 2020), and are an important source of gene family expansion. After multiple sequence alignment of sequences in synteny blocks within *Q. acutissima* and other species, the synteny analysis showed that *Q. acutissima* had a 1:1 syntenic relationship with other Fagaceae, and there was little rearrangement of chromosomes, which indicated that the evolution of Fagaceae was very conserved and no independent WGD events occurred in *Q. acutissima* (**Figure 3**, **Figures S4-S11**).

## Discussion

*Q. acutissima*, Fagaceae, is an economically and ecologically important tree species with wide distribution in China (Li et al., 2018; Zhang et al., 2020). Here, we generated a *Q. acutissima* genome at the chromosome-level. The assembled genome size is approximately 956.9 Mb, which is larger than the genome we assessed using the *K*-mer method, this may be due to the presence of chimerism in our assembly. The development of PacBio sequencing has resulted in a considerable increase in contig N50 sizes compared to previous sequencing technologies (Wei et al., 2020). The assemble length of contig N50 sizes can represent the genome assembling quality (Yang et al., 2021), consequently, our genome has high assembly contiguity. High heterozygosity and repetition rates are responsible for the inability to assemble high-quality genomes (Gao et al., 2020; Wang et al., 2021). *Q. acutissima* heterozygous rate was 2.77%, which is higher than that of *Q. lobata* (1.25%) (Sork et al., 2016) and *Q. suber* (1.62%) (Ramos et al., 2018). It is worth noting that 98% of complete BUSCO core genes were detected in the assembled genome, which is higher than that of *Q. lobata* (94%) (Sork et al., 2016) and comparable to *Q. suber* genome (97%) (Ramos et al., 2018). In summary, *Q. acutissima* assembly is relatively accurate and complete, which will provide a valuable genome resource for understanding the species evolution and enhance its genetic improvement.

The genus *Quercus* (Fagaceae), which includes 400-500 species, is distributed in Asia, Africa, Europe, and North America (Simeone et al., 2016; Bent, 2020). As a member in this genus, *Q. acutissima* genome information can fill genome research gap and promote the species evolutionary biology research. Following the statistical analysis of repeat in the genome, we found that the repeat regions accounted for 57.13%, the numbers of repetitive and ncRNA sequences were relatively high in *Q. acutissima* compared with other *Quercus* species. To understand the evolutionary development of *Q. acutissima*, we analyzed its evolution and divergence times. The syntenic analysis indicated that *Q. acutissima* did not experienced a recent WGD event. In plants, WGD events can lead to genome size variation, gene family expansion, chromosomal rearrangement, and species evolution (El Baidouri and Panaud, 2013; Wang et al., 2021). We found high collinearity relationship between *Q. acutissima* and *Q. variabilis* chromosomes, suggesting the conservative nature of their karyotypes.

In summary, we obtained high-quality *Q. acutissima* genome sequences using Pacbio, Hi-C and Illumina reads. The development of sequencing technologies, analytical methods, and statistical algorithms continue to promote the efficiency and accuracy of genome sequencing and assembly (Xue et al., 2020; Wei et al., 2020; Wu et al., 2020). *Q. acutissima* genome includes high quality chromosomal-level assembly and many important genes, offering novel insights into genome evolution, functional innovation, and key regulatory pathways in wood formation and production of high-value metabolites, and providing excellent genetic resources for comparative genome studies among *Quercus* species.

## Data availability statement

The data presented in the study are deposited in the CNGB Sequence Archive (CNSA, https://db.cngb.org/cnsa/) of China National GeneBank DataBase (CNGBdb) repository, accession number CNP0003530, CNP0002992.

## Author contributions

WeiL and WenL designed and supervised the study. DL, XX, BT, CZ, and KQ collected the samples and extracted the genomic DNA and RNA. DL, CZ, KQ, HG and ZZ performed genome assembly and bioinformatics analysis. YE did English editing and retouching. DL wrote the original manuscript. WeiL and WenL reviewed and edited this manuscript. All authors read and approved the final manuscript.

## Funding

This research was funded by the Collection and arrangement of genetic resources and genetic diversity evaluation of *Quercus acutissima* of Biosafety and Genetic Resources Management Project of State Forestry and Grassland Administration, grant number KJZXSA202111; ‘Collection, Conservation, and Accurate Identification of Forest Tree Germplasm Resources’ of Shandong Provincial Agricultural Elite Varieties Project, grant number 2019LZGC018; Project of National Forest Germplasm Resources Sharing Service Platform Construction and Operation, grant number 2005-DKA21003.

## Acknowledgments

We are grateful for the generous grant from the National Engineering Research Center of Tree Breeding and Ecological Restoration and Shandong Provincial Center of Forest and Grass Germplasm Resources that made this work possible.

## Conflict of interest

The authors declare that the research was conducted in the absence of any commercial or financial relationships that could be construed as a potential conflict of interest.

## Publisher’s note

All claims expressed in this article are solely those of the authors and do not necessarily represent those of their affiliated organizations, or those of the publisher, the editors and the reviewers. Any product that may be evaluated in this article, or claim that may be made by its manufacturer, is not guaranteed or endorsed by the publisher.
