# Supplementary material for: A high-quality genome assembly and annotation of Quercus acutissima Carruth
Source: Front Plant Sci. 2022 Nov 24;13:1068802. doi: 10.3389/fpls.2022.1068802 (PMC9729791; doi:10.3389/fpls.2022.1068802)
Supplement: Supplementary file 1 [file DataSheet_1.docx]

Supplementary Material

# Supplementary Figures and Tables

## Supplementary Figures


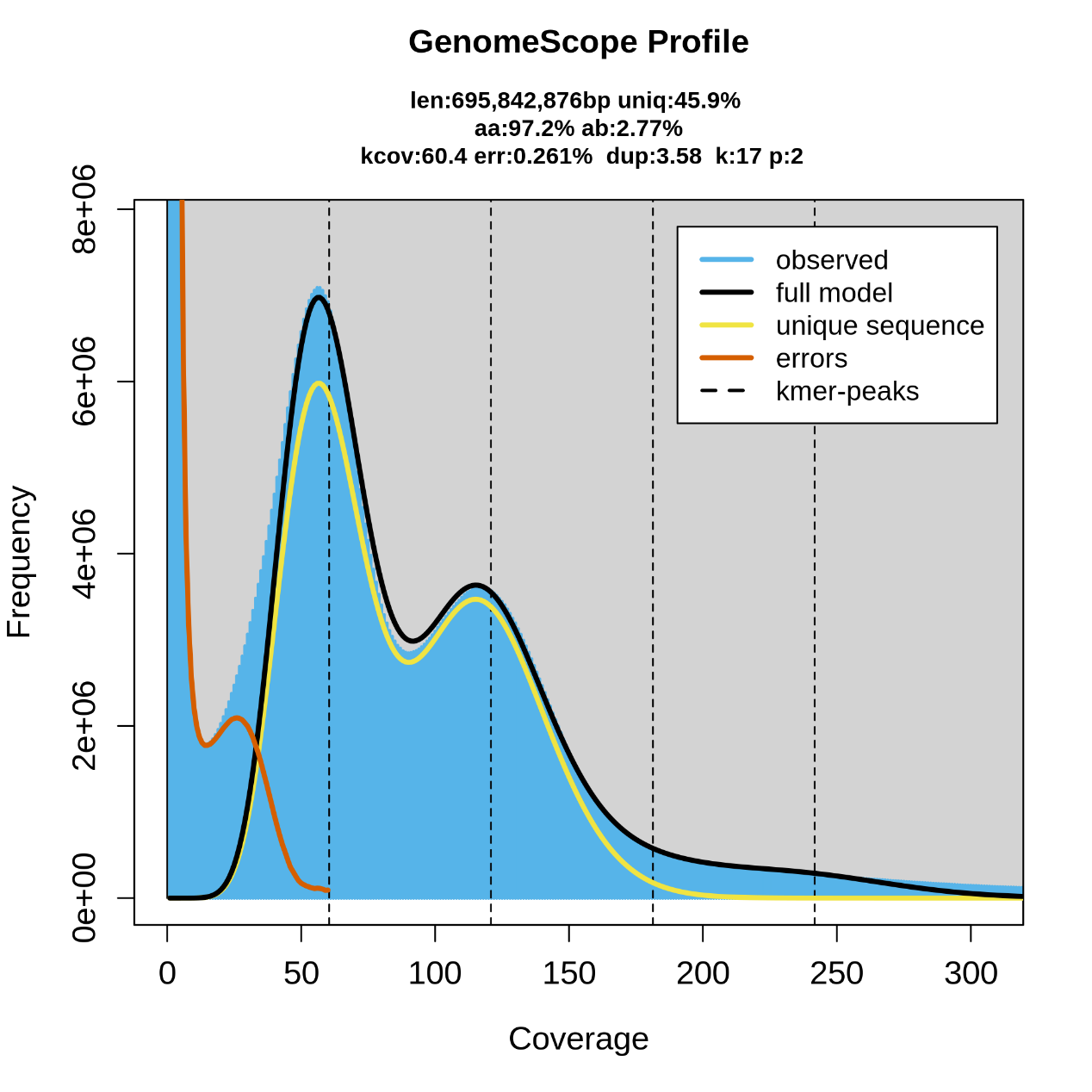


**Figure S1.** K-mer depth frequency curve. X- and Y-axes indicate the depth of K-mer (the number of occurrences of K-mer). and the number of K-mer corresponding to the depth as a percentage of the total number of K-mer, respectively.


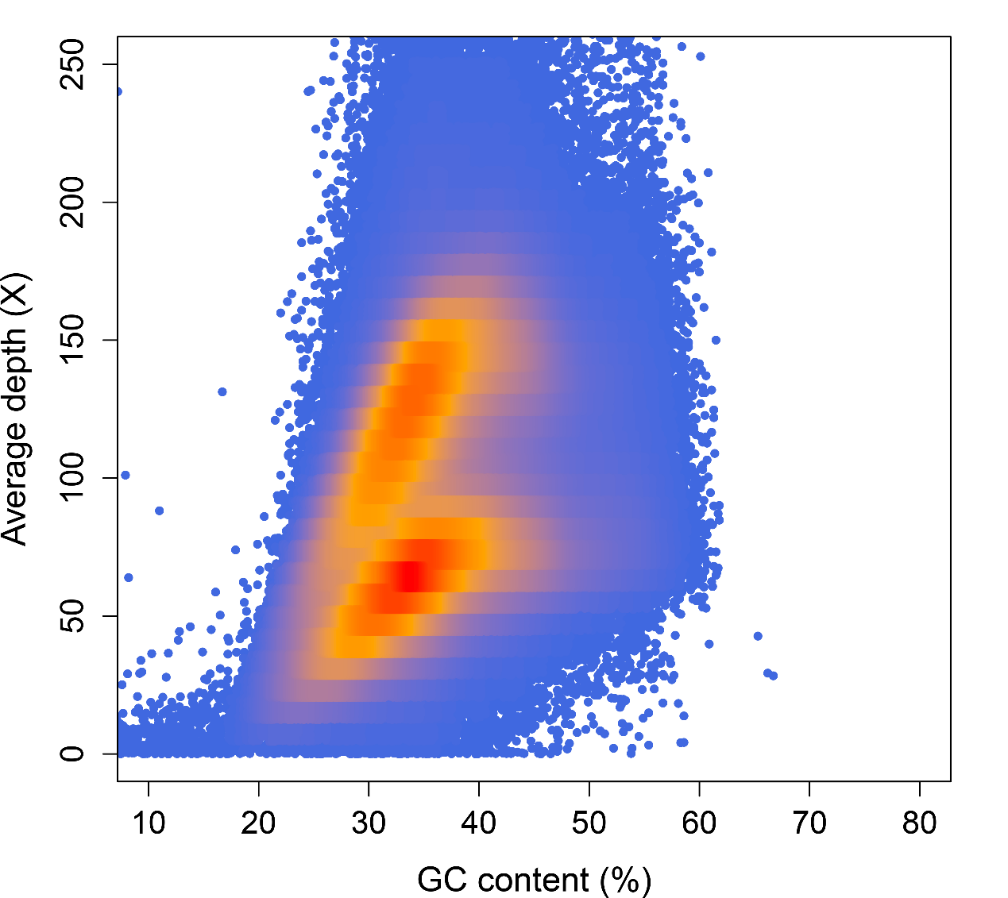


**Figure S2.** Scatter map of GC depth in genome assembly. X and Y-axes indicates GC content and Depth, respectively, these two values are counted sequentially in a 10 kb window.


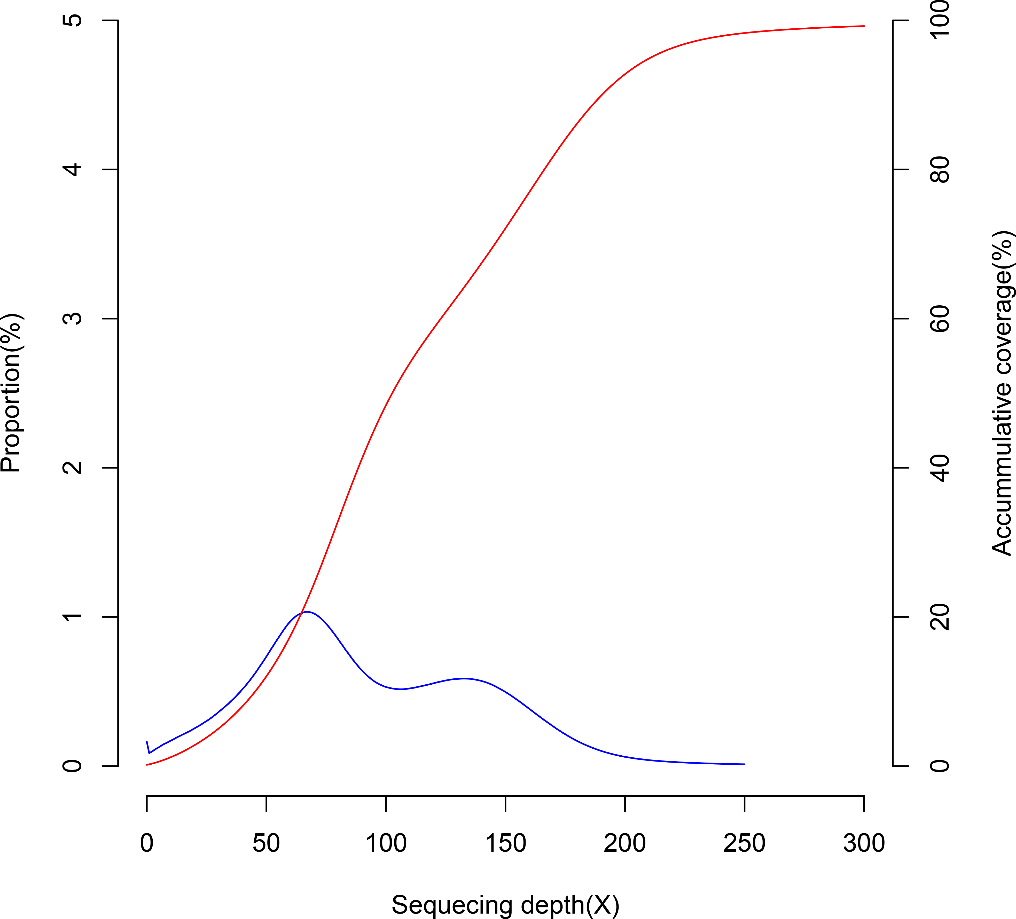


**Figure S3.** Read coverage depth distribution and integrity of genome coverage. The Blue and red lines indicate depth distribution curve and accumulative coverage curve, respectively.

**
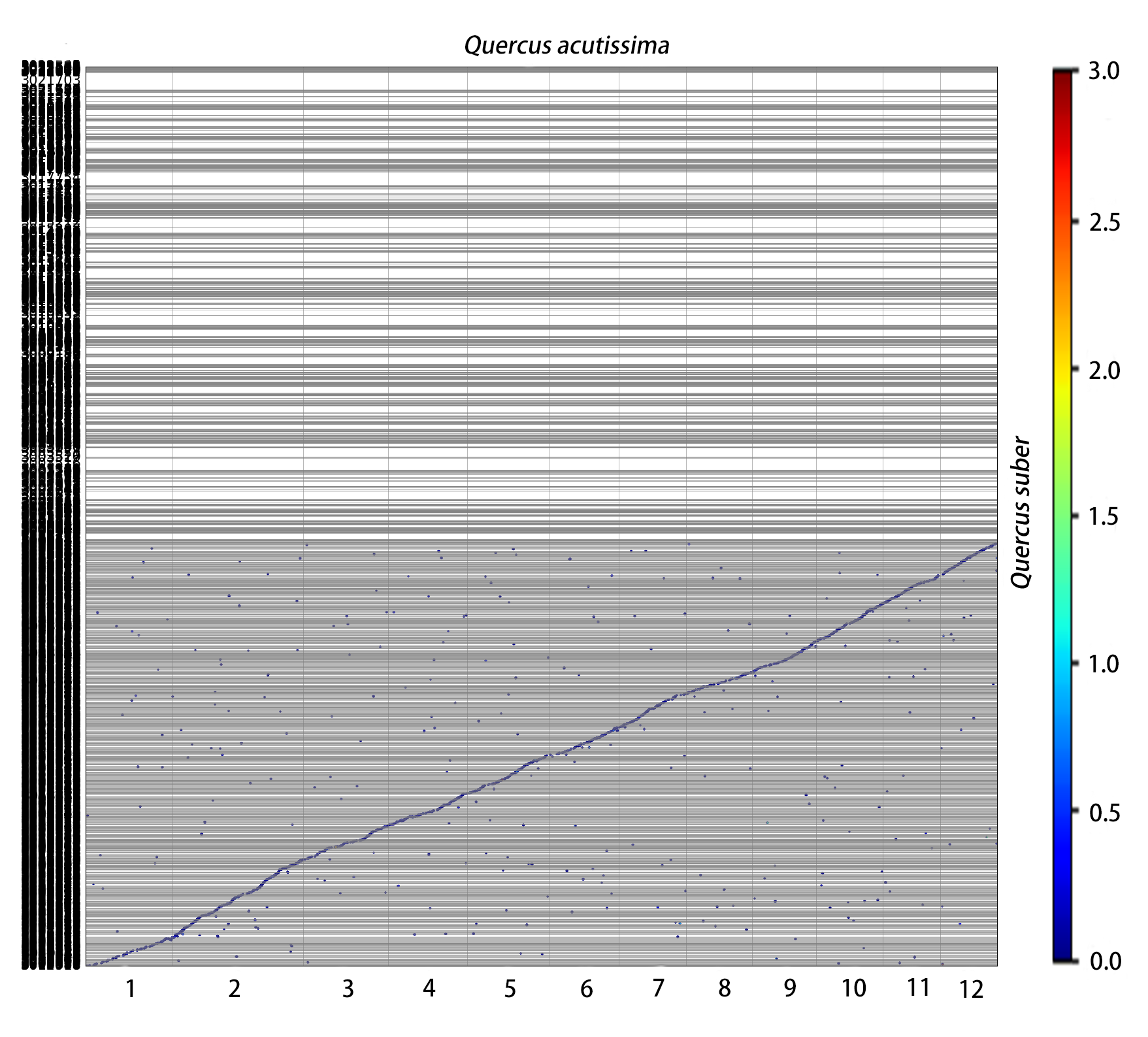
Figure S4.** Syntenic dot plot between the *Q. acutissima* and *Q. suber* genome.

**
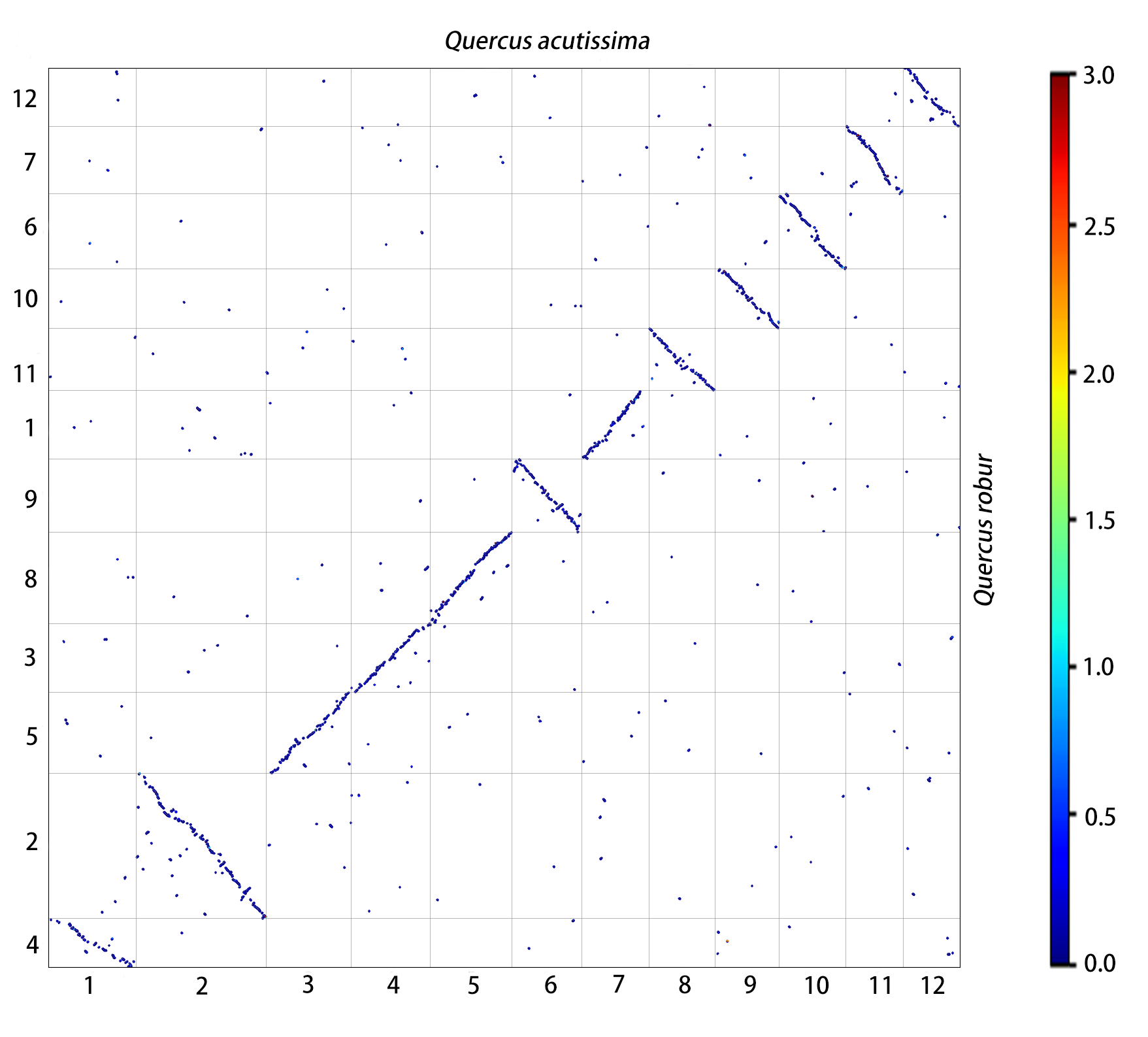
**

**Figure S5.** Syntenic dot plot between the *Q. acutissima* and *Q. robur* genome.


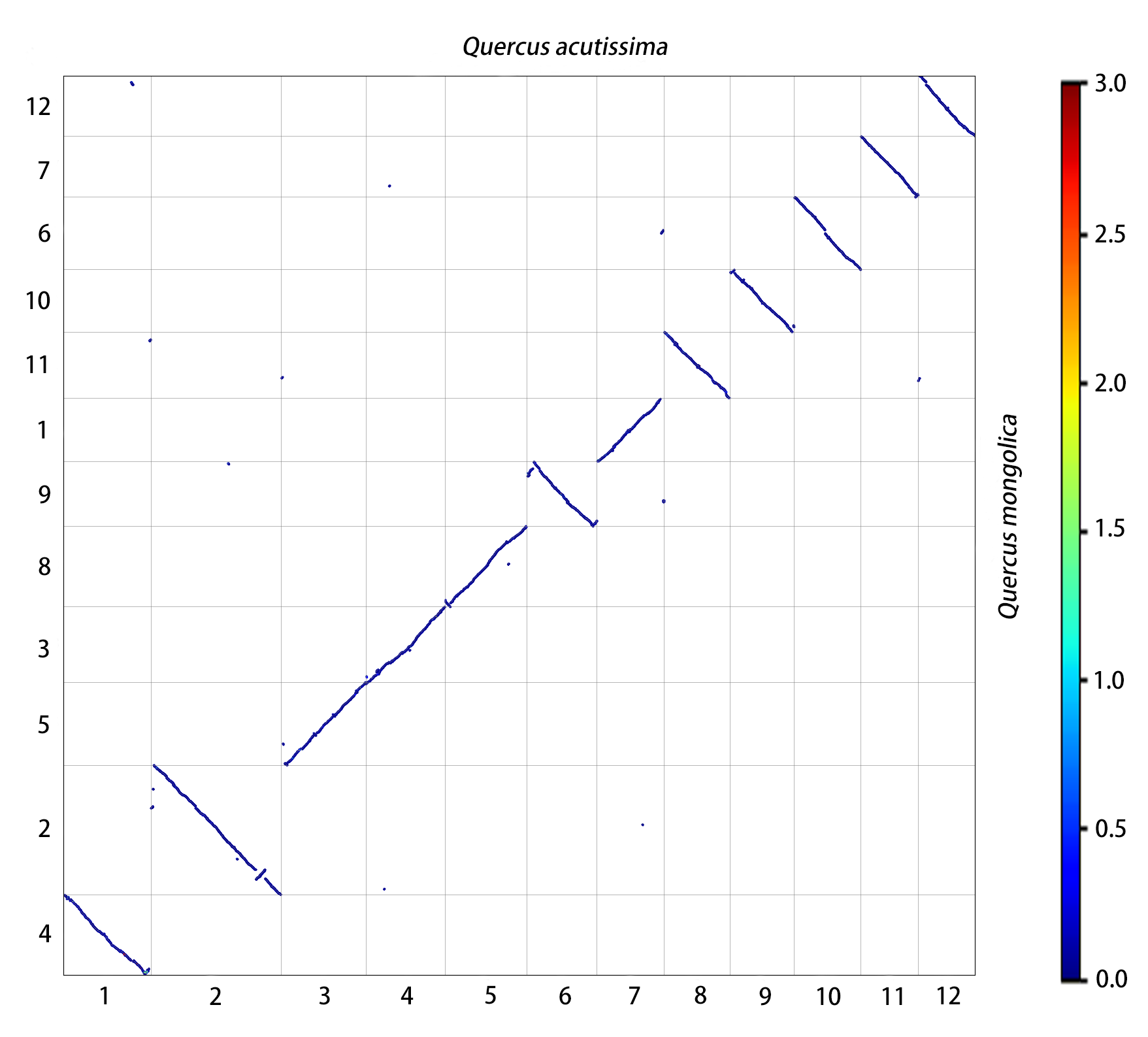


**Figure S6.** Syntenic dot plot between the *Q. acutissima* and *Q. mongolica* genome.


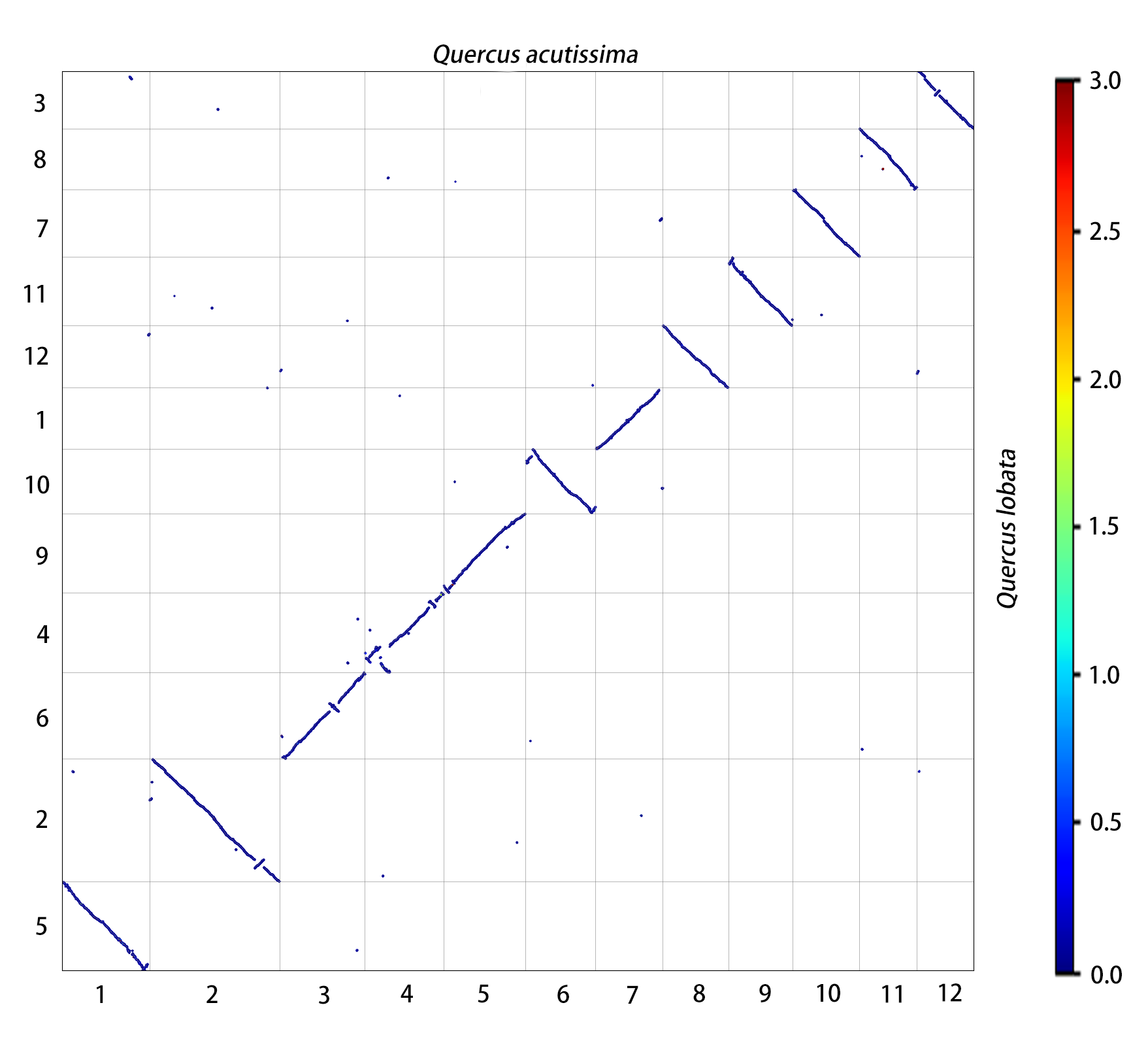


**Figure S7.** Syntenic dot plot between the *Q. acutissima* and *Q. lobata* genome.


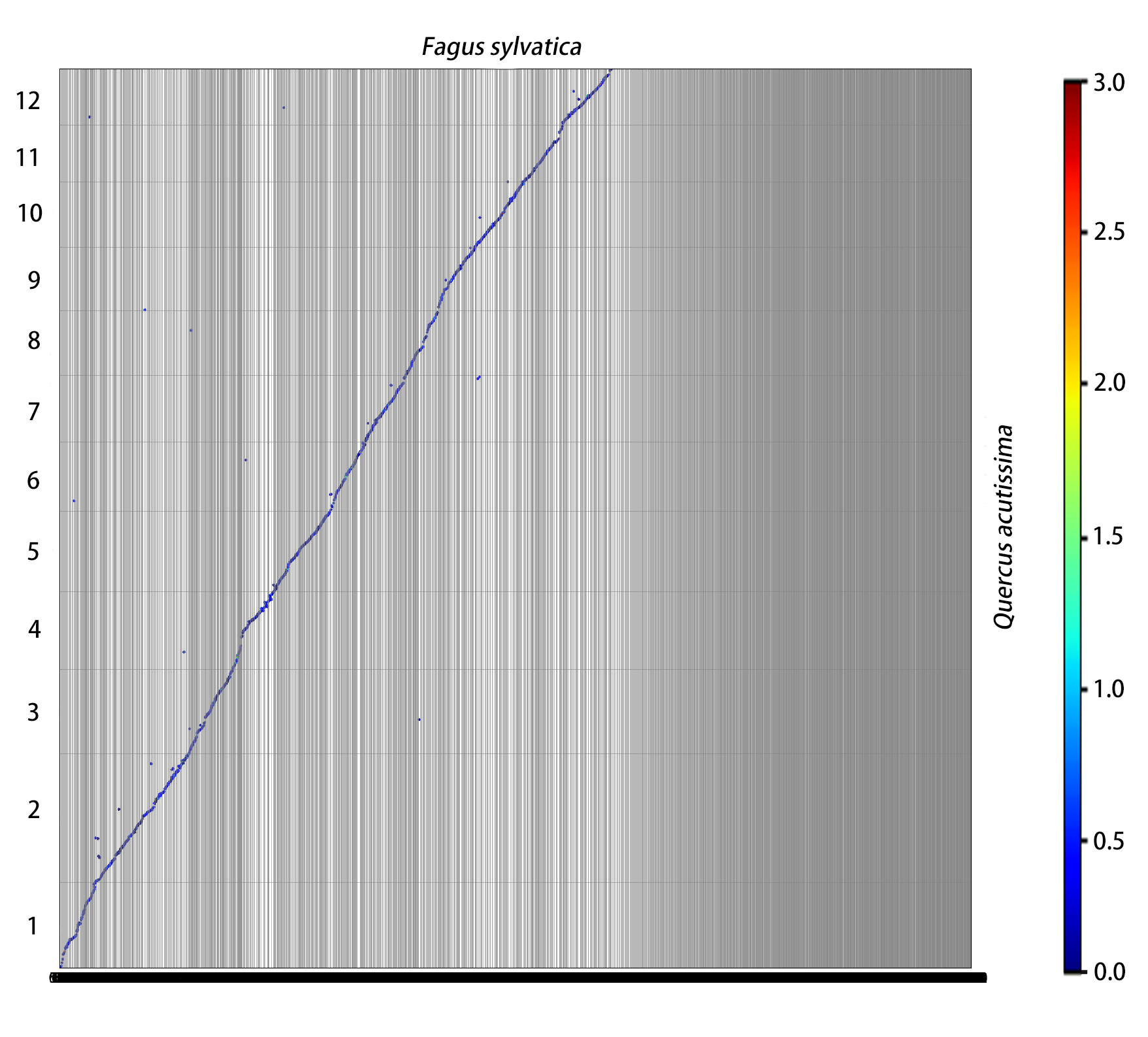
**Figure S8.** Syntenic dot plot between the *Q. acutissima* and *F. sylvatica* genome.


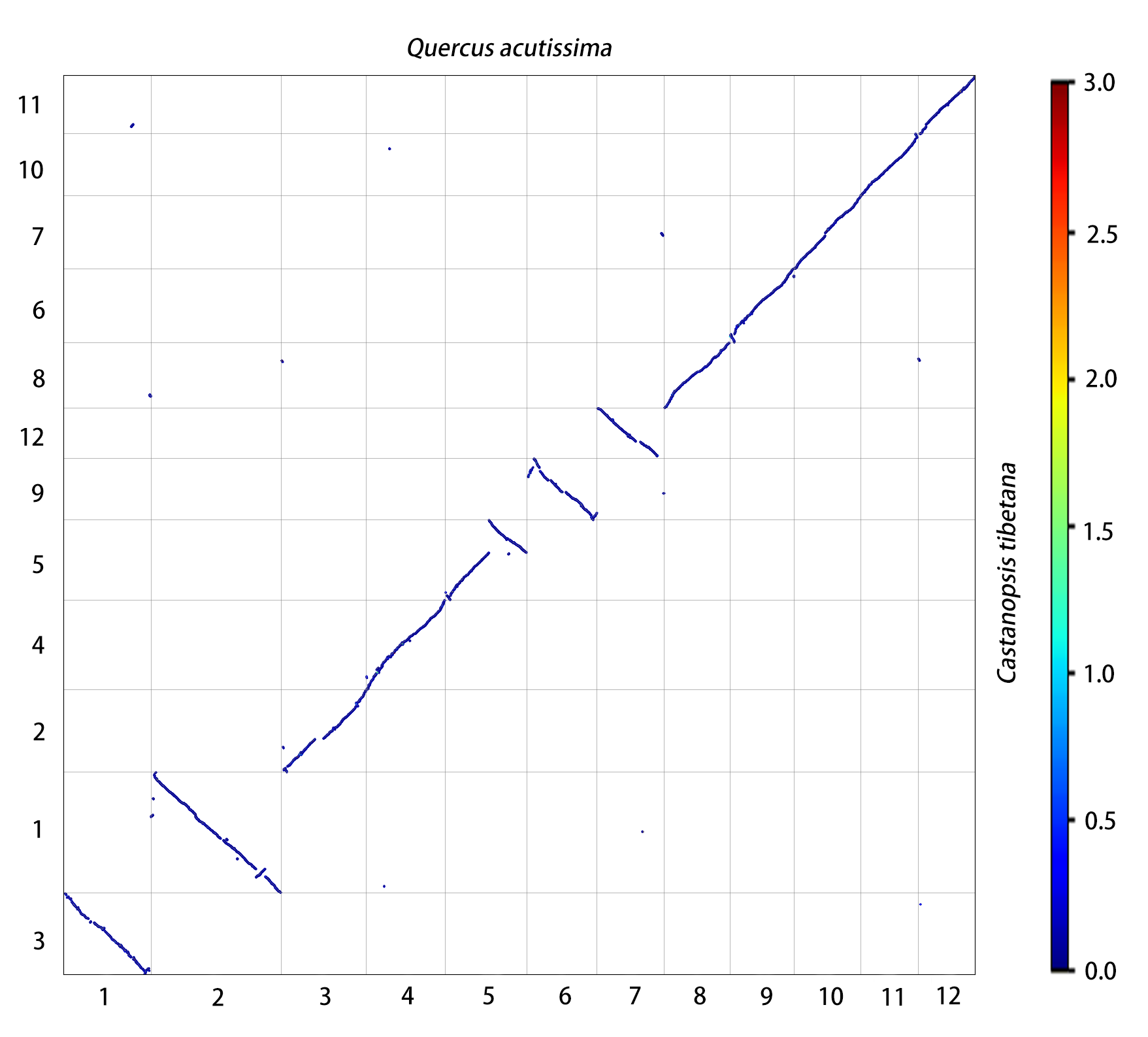
**Figure S9.** Syntenic dot plot between the *Q. acutissima* and *C. tibetana* genome.


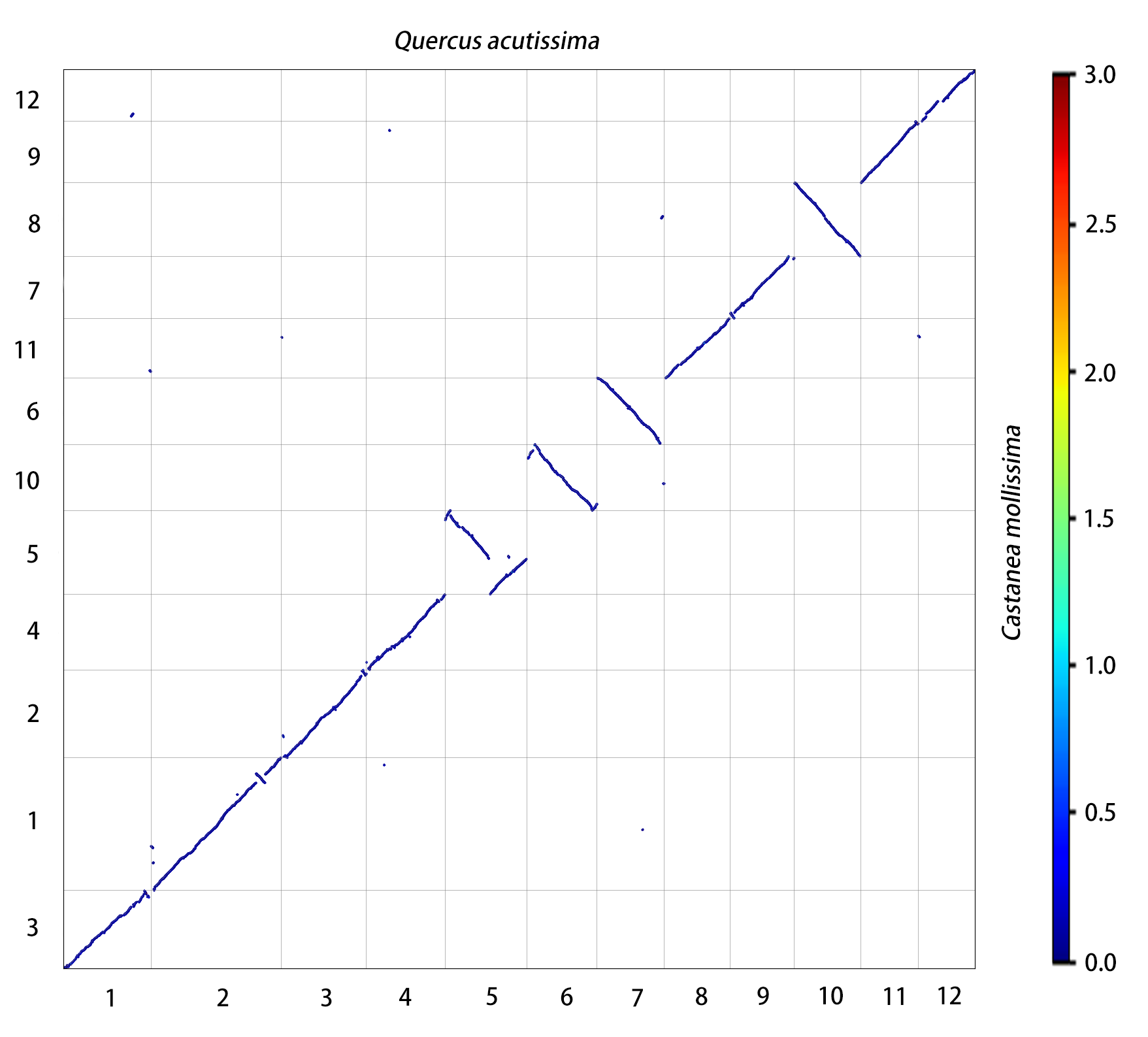


**Figure S10.** Syntenic dot plot between the *Q. acutissima* and *C. mollissima* genome.


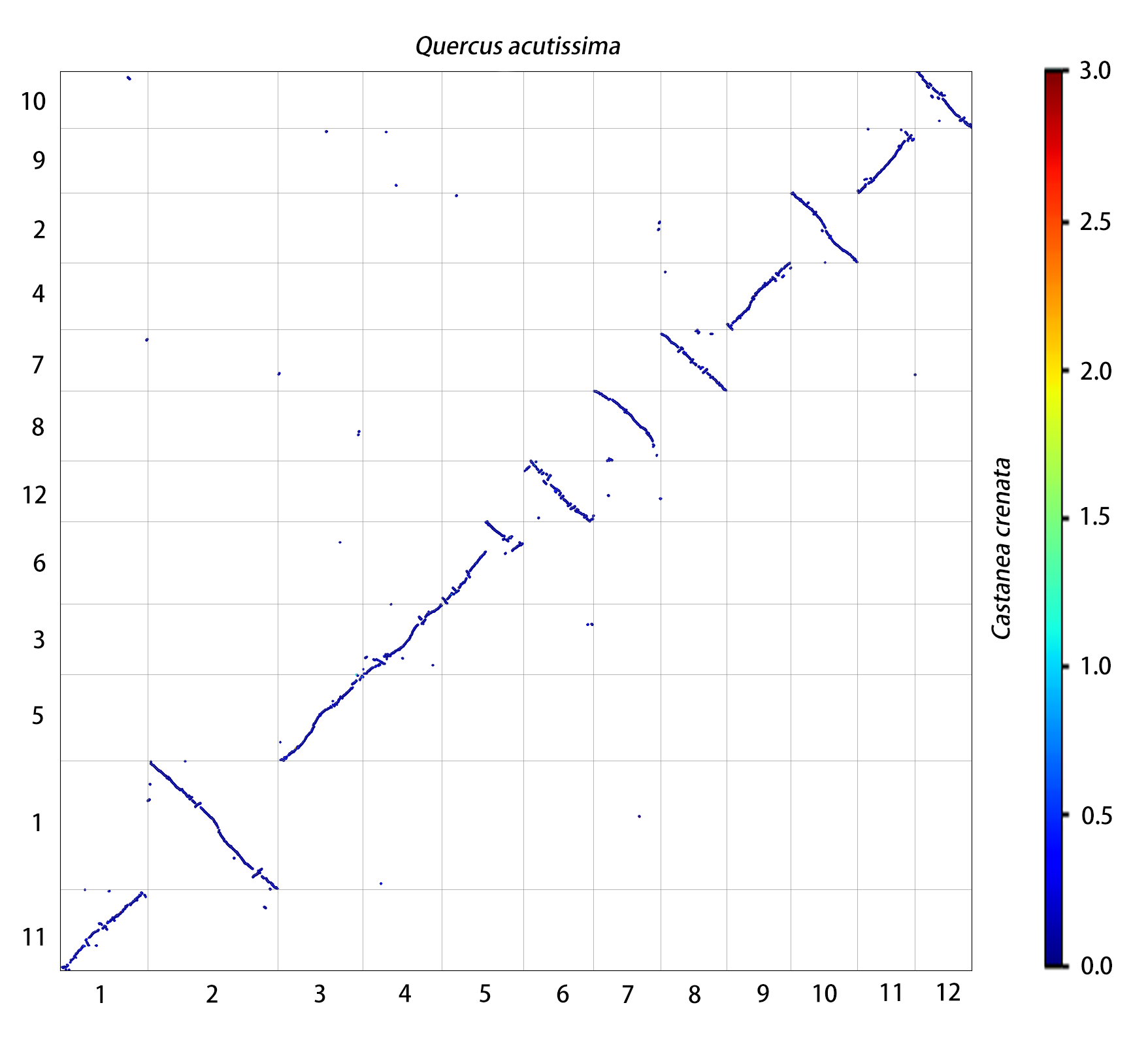


**Figure S11.** Syntenic dot plot between the *Q. acutissima* and *C. crenata* genome.

## Supplementary Tables

**Table S1.** PacBio Sequel sequencing data statistics.

| **Index** | **PacBio** |
| --- | --- |
| Subreads number | 8,972,662 |
| Subreads Average Mean Length(bp) | 17,209.06 |
| Subreads N50(bp) | 24,256 |
| Subreads Read Base(bp) | 154,411,063,662 |
| GC mean | 0.38 |

**Table S2.** BUSCO assessment statistics.

| **Items** | **Number** | **Percent (%)** |
| --- | --- | --- |
| Complete BUSCOs (C) | 1,347 | 98.0 |
| Complete and single-copy BUSCOs (S) | 1,109 | 80.7 |
| Complete and duplicated BUSCOs (D) | 238 | 17.3 |
| Fragmented BUSCOs (F) | 8 | 0.6 |
| Missing BUSCOs (M) | 20 | 1.4 |
| Total BUSCO groups searched | 1,375 | 100.0 |

**Table S3.** Base content statistics.

| **Items** | **Number** | **Percent (%)** |
| --- | --- | --- |
| A | 309,992,182 | 32.39% |
| T | 310,260,412 | 32.42% |
| C | 168,427,959 | 17.60% |
| G | 168,256,922 | 17.58% |
| N | 0 | 0.00% |
| GC | 336,684,881 | 35.18% |
| Total Genome base | 956,937,475 | - |

**Table S4.** Identity assessment statistics.

| **Sample** | **ML** |
| --- | --- |
| Clean Reads | 669,368,636 |
| Clean Bases | 100,405,295,400 |
| Mapped Reads | 664,600,033 |
| Mapped Reads Rate (%) | 99.29 |
| Mapped Bases | 98,226,520,915 |
| Mapped Bases Rate (%) | 97.83 |
| Mean Depth | 99.40 |
| Coverage Rate (%) | 99.84 |

**Table S5.** Repeat sequence statistics.

| **Type** | **Repeat length(bp)** | **% of genome** |
| --- | --- | --- |
| RepeatMasker | 95,429,294 | 9.97 |
| ProteinMask | 146,533,493 | 15.31 |
| De novo | 496,425,213 | 51.88 |
| Trf | 47,779,176 | 4.99 |
| Total | 546,670,373 | 57.13 |

**Table S6.** Repeated sequence classification statistics.

| **Class** |  | **RepeatMasker TEs** | | **RepeatProteinMask TEs** | | **RepeatModeler TEs** | | **Combined TEs** |
| --- | --- | --- | --- | --- | --- | --- | --- | --- |
| Type | Length(bp) | % in genome | Length(bp) | % in genome | Length(bp) | % in genome | Length(bp) | % in genome |
| DNA | 11,563,703 | 1.21 | 2,093,933 | 0.22 | 15,257,137 | 1.59 | 22,456,692 | 2.35 |
| LINE | 13,179,085 | 1.38 | 21,490,359 | 2.25 | 37,243,240 | 3.89 | 41,329,336 | 4.32 |
| SINE | 101,971 | 0.01 | 0 | 0 | 0 | 0 | 101,971 | 0.01 |
| LTR | 68,664,713 | 7.18 | 86,138,668 | 9.00 | 205,808,272 | 21.51 | 220,739,375 | 23.07 |
| Unknown | 129,800 | 0.01 | 63 | 0 | 241,576,948 | 25.24 | 241,681,590 | 25.26 |
| Other | 3,086,737 | 0.32 | 37,032,277 | 3.87 | 6,385,605 | 0.67 | 44,694,294 | 4.67 |
| Total | 95,429,294 | 9.97 | 146,533,493 | 15.31 | 496,425,213 | 51.88 | 532,334,632 | 55.63 |

**Table S7.** Number of annotated genes.

| **Database** | **Count** | **Percentage(%)** |
| --- | --- | --- |
| BLASTP | 24,679 | 82.57 |
| BLASTX | 24,439 | 81.77 |
| GO | 24,655 | 82.49 |
| KO | 8,890 | 29.74 |
| Map | 5,608 | 18.76 |
| NR | 29,836 | 99.82 |
| NT | 22,005 | 73.62 |
| PFAM | 24,438 | 81.76 |
| eggNOG | 20,784 | 69.54 |
| Total_annotated genes | 24,689 | 82.60 |
| Total_unigene | 29,889 | 100.00 |

**Table S8.** Quantitative statistics of gene clustering into families.

| **Species** | **Number of genes** | **Number of genes in orthogroups** | **Number of unassigned genes** | **Percentage of genes in orthogroups** | **Percentage of unassigned genes** | **Number of orthogroups containing species** | **Percentage of orthogroups containing species** | **Number of species-specific orthogroups** | **Number of genes in species-specific orthogroups** | **Percentage of genes in species-specific orthogroups** |
| --- | --- | --- | --- | --- | --- | --- | --- | --- | --- | --- |
| *B. pendula* | 23,527 | 20,025 | 3,502 | 85.1 | 14.9 | 13,700 | 48.4 | 15 | 39 | 0.2 |
| *C. viminea* | 26,621 | 24,364 | 2,257 | 91.5 | 8.5 | 14,434 | 51 | 20 | 80 | 0.3 |
| 1. *illinoinensis* | 32,267 | 29,900 | 2,367 | 92.7 | 7.3 | 15,305 | 54.1 | 8 | 47 | 0.1 |
| *C. crenata* | 69,980 | 47,208 | 22,772 | 67.5 | 32.5 | 18,392 | 65 | 181 | 530 | 0.8 |
| *C. mollissima* | 33,597 | 32,707 | 890 | 97.4 | 2.6 | 16,880 | 59.6 | 12 | 46 | 0.1 |
| *C. tibetana* | 40,937 | 37,144 | 3,793 | 90.7 | 9.3 | 18,351 | 64.8 | 29 | 101 | 0.2 |
| *C. mandshurica* | 28,409 | 25,353 | 3,056 | 89.2 | 10.8 | 15,538 | 54.9 | 32 | 118 | 0.4 |
| *C. paliurus* | 56,403 | 39,501 | 16,902 | 70 | 30 | 13,900 | 49.1 | 177 | 828 | 1.5 |
| *F. sylvatica* | 62,012 | 41,763 | 20,249 | 67.3 | 32.7 | 16,527 | 58.4 | 387 | 1,546 | 2.5 |
| *J. regia* | 31,523 | 30,599 | 924 | 97.1 | 2.9 | 14,448 | 51 | 10 | 34 | 0.1 |
| *M. rubra* | 29,351 | 25,620 | 3,731 | 87.3 | 12.7 | 14,083 | 49.7 | 74 | 473 | 1.6 |
| *Q. acutissima* | 29,889 | 29,204 | 685 | 97.7 | 2.3 | 15,802 | 55.8 | 10 | 27 | 0.1 |
| *Q. lobata* | 36,705 | 35,724 | 981 | 97.3 | 2.7 | 16,775 | 59.3 | 17 | 64 | 0.2 |
| *Q. mongolica* | 36,553 | 33,507 | 3,046 | 91.7 | 8.3 | 17,829 | 63 | 30 | 122 | 0.3 |
| *Q. robur* | 25,808 | 24,789 | 1,019 | 96.1 | 3.9 | 13,282 | 46.9 | 6 | 13 | 0.1 |
| *Q. suber* | 79,281 | 46,952 | 32,329 | 59.2 | 40.8 | 20,534 | 72.5 | 52 | 176 | 0.2 |
| *Q. variabilis* | 32,466 | 28,402 | 4,064 | 87.5 | 12.5 | 16,048 | 56.7 | 11 | 74 | 0.2 |
| *V. vinifera* | 31,845 | 25,227 | 6,618 | 79.2 | 20.8 | 15,153 | 53.5 | 69 | 297 | 0.9 |

**Table S9.** Expansions and contractions statistics.

| **Species** | **Expansions** | | | **Contractions** | | | **No Change** | **Avg. Expansion** |
| --- | --- | --- | --- | --- | --- | --- | --- | --- |
|  | **Families** | **Genes** | **Gene Gain/ Family** | **Families** | **Genes** | **Gene Loss/ Family** |  |  |
| 1. *sylvatica* | 3,209 | 10,810 | 3.37 | 3,283 | 3,560 | 1.08 | 11,292 | 0.40767 |
| 1. *regia* | 1,782 | 3,413 | 1.92 | 2,519 | 3,459 | 1.37 | 13,483 | -0.00258659 |
| 1. *suber* | 4,337 | 10,713 | 2.47 | 1,215 | 1,452 | 1.20 | 12,232 | 0.520749 |
| 1. *crenata* | 1,921 | 8,518 | 4.43 | 1,866 | 2,229 | 1.19 | 13,997 | 0.353632 |
| 1. *pendula* | 703 | 1,395 | 1.98 | 3,566 | 4,302 | 1.21 | 13,515 | -0.163462 |
| 1. *paliurus* | 4,538 | 11,442 | 2.52 | 4,098 | 5,166 | 1.26 | 9,148 | 0.352901 |
| *Q. acutissima* | 2,390 | 3,619 | 1.51 | 3,897 | 5,713 | 1.47 | 11,497 | -0.117746 |
| *V. vinifera* | 1,909 | 3,373 | 1.77 | 4,472 | 5,145 | 1.15 | 11,403 | -0.0996401 |
| *C. viminea* | 1,332 | 2,985 | 2.24 | 2,458 | 2,737 | 1.11 | 13,994 | 0.0139451 |
| *Q. robur* | 1,378 | 3,251 | 2.36 | 6,060 | 7,925 | 1.31 | 10,346 | -0.262821 |
| *C. mandshurica* | 1,397 | 2,701 | 1.93 | 1,069 | 1,549 | 1.45 | 15,318 | 0.0647773 |
| *M. rubra* | 2,230 | 4,115 | 1.85 | 3,540 | 4,202 | 1.19 | 12,014 | -0.00489204 |
| *C. tibetana* | 3,015 | 6,608 | 2.19 | 2,226 | 3,236 | 1.45 | 12,543 | 0.189609 |
| *Q. mongolica* | 2,358 | 4,330 | 1.84 | 1,599 | 2,885 | 1.80 | 13,827 | 0.0812528 |
| *C. mollissima* | 1,052 | 2,207 | 2.10 | 1,919 | 2,897 | 1.51 | 14,813 | -0.0387989 |
| *Q. lobata* | 2,325 | 5,745 | 2.47 | 2,053 | 2,405 | 1.17 | 13,406 | 0.187809 |
| *C. illinoinensis* | 1,199 | 2,081 | 1.74 | 1,060 | 1,236 | 1.17 | 15,525 | 0.0475146 |
| *Q. variabilis* | 980 | 2,714 | 2.77 | 5,085 | 7,715 | 1.52 | 11,719 | -0.281208 |

**
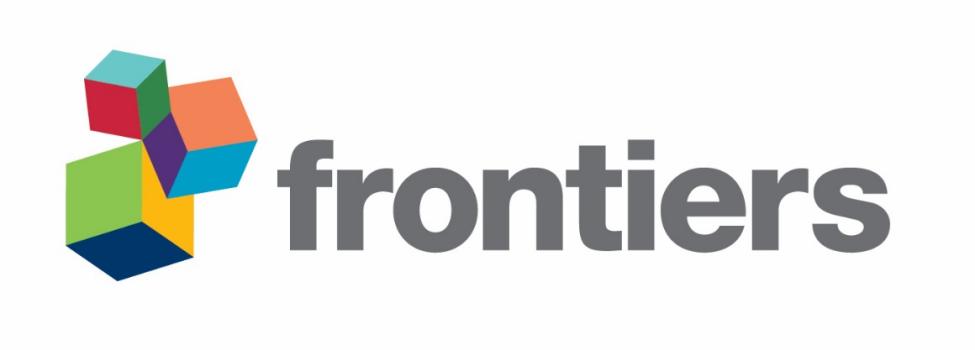
**
